# Supplementary material for: Relating Global and Local Connectome Changes to Dementia and Targeted Gene Expression in Alzheimer's Disease
Source: Front Hum Neurosci. 2021 Dec 17;15:761424. doi: 10.3389/fnhum.2021.761424 (PMC8734427; doi:10.3389/fnhum.2021.761424)
Supplement: Supplementary file 1 [file Data_Sheet_1.pdf]

Supplementary Materials for: Relating Global and Local Connectome Changes to Dementia and Targeted Gene Expressions in Alzheimer's Disease

1 SUPPLEMENTARY DATA

1.1 Figures

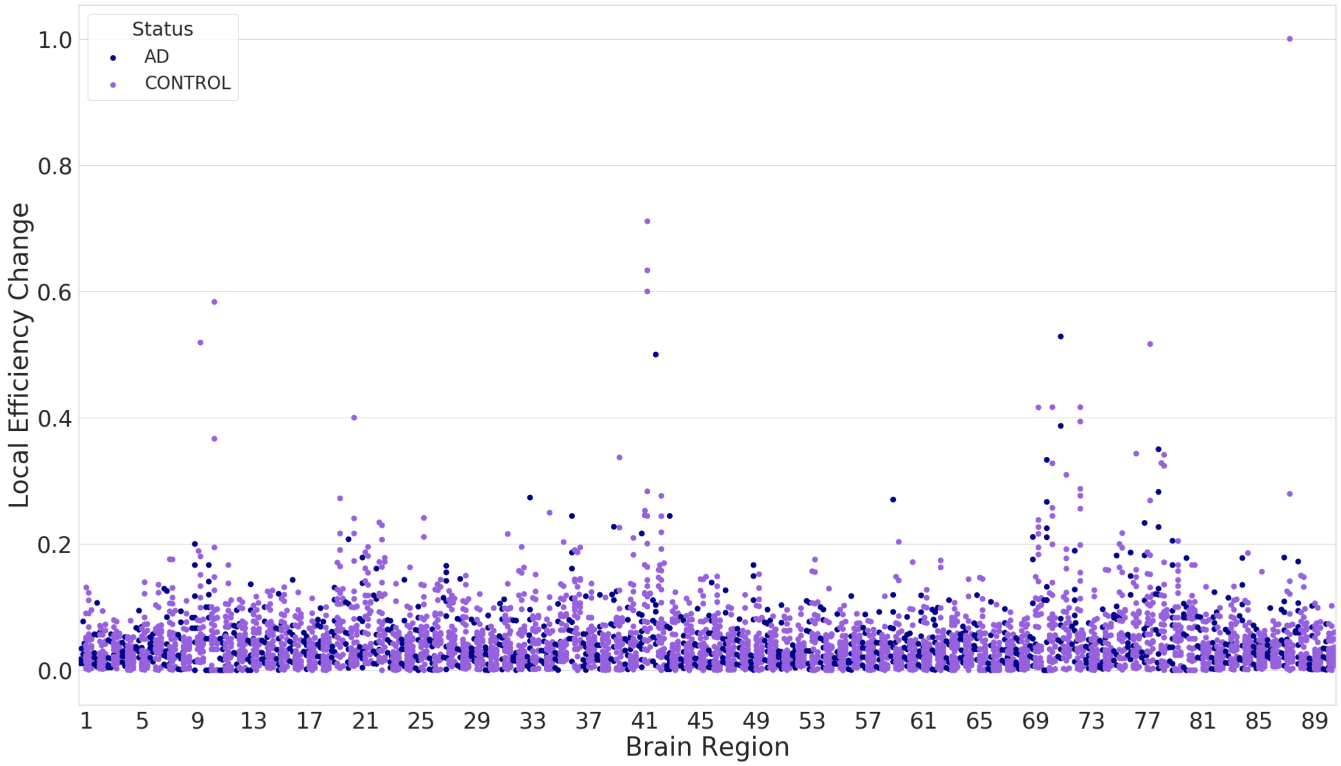

Figure S1: The figure shows the distribution of the absolute differences between the baseline and follow-up measures of local efficiency of the AD (blue) vs controls (purple), along the 90 AAL brain regions.

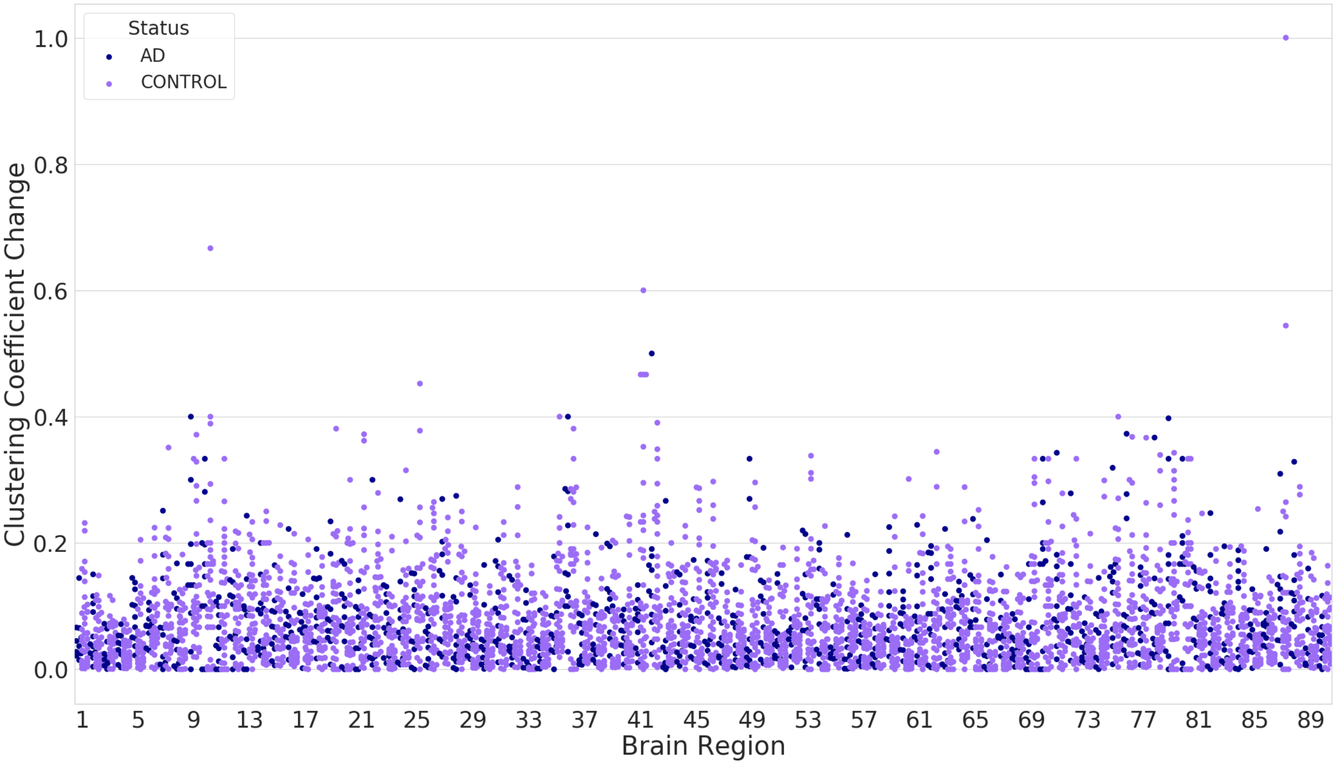

Figure S2: The figure shows the distribution of the absolute differences between the baseline and follow-up measures of clustering coefficient of the AD (blue) vs controls (purple), along the 90 AAL brain regions.

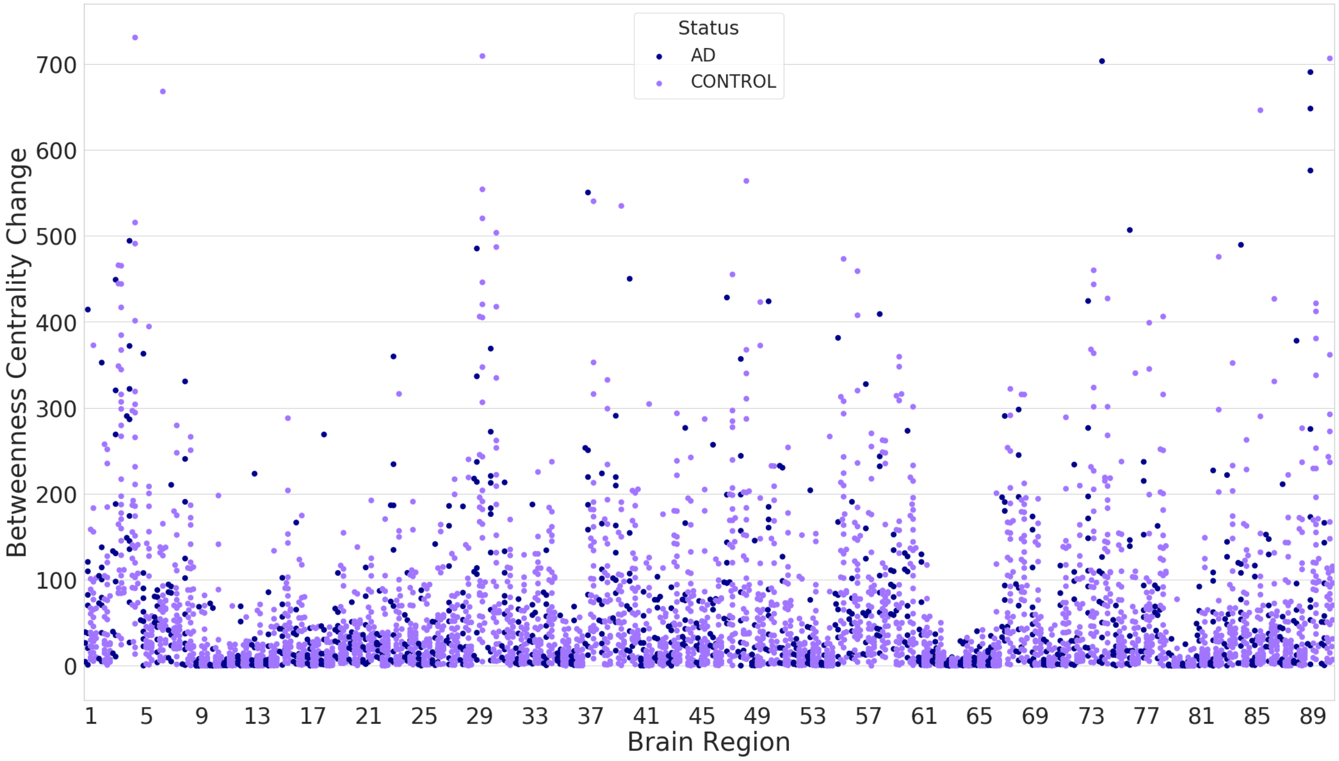

Figure S3: The figure shows the distribution of the absolute differences between the baseline and follow-up measures of betweenness centrality of the AD (blue) vs controls (purple), along the 90 AAL brain regions.

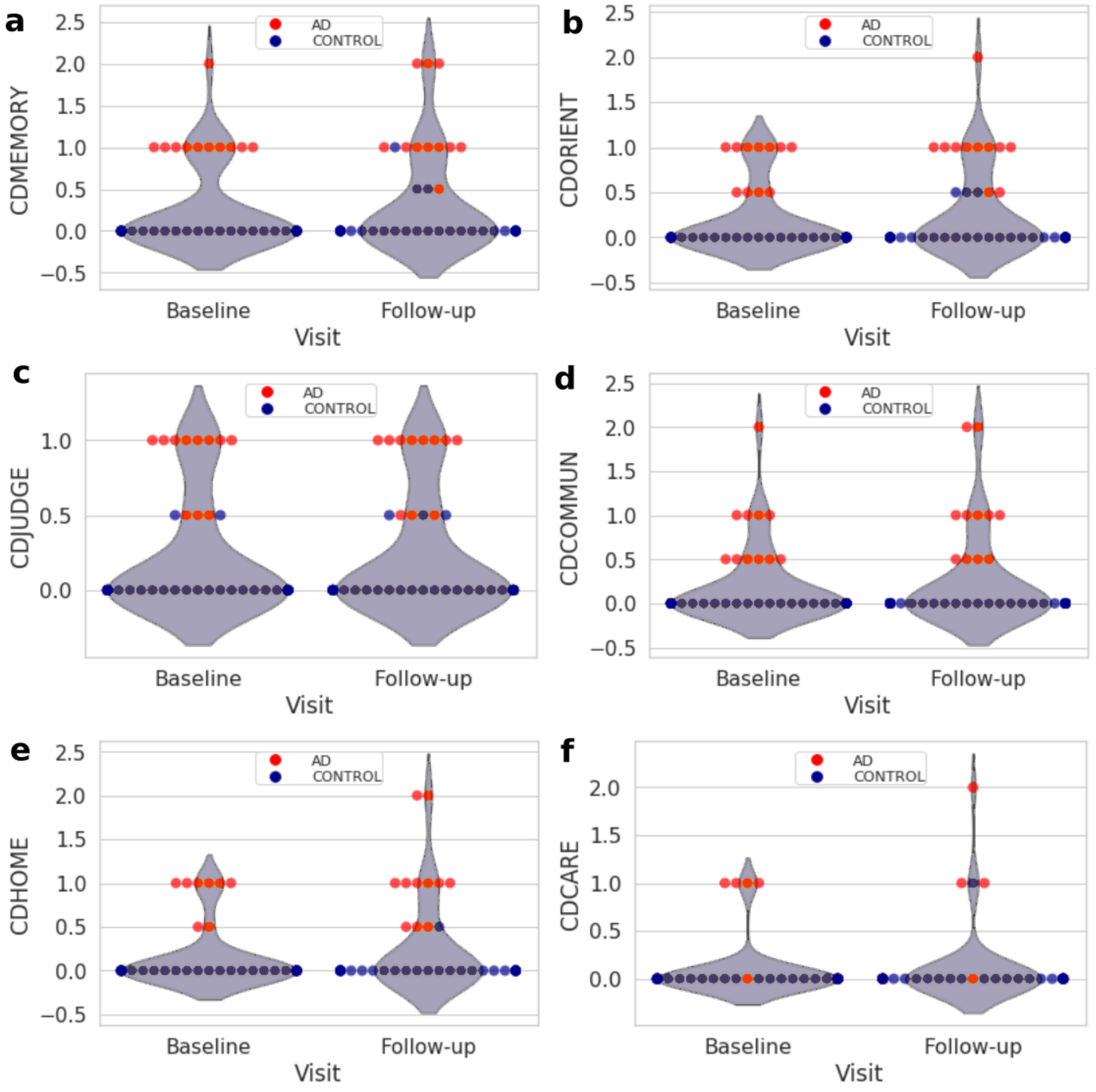

Figure S4: Violin plots to illustrate the CDR scores (either 0: None, 0.5: very mild, 1: mild, 2: moderate or 3: severe) in the baseline (left violin plot) and follow-up (right violin plot) visits, for AD (red dots) and controls (blue dots). The more dots moved to the higher scores from Baseline to Follow-up, the more patients worsened their disabilities. The memory (CDMEMORY; a) and orientation (CDORIENT; b) scores are represented by the top sub-figures, judgment and problem solving (CDJUDGE; c) and community affairs (CDCOMMUN; d) are the middle sub-figures, while home and hobbies (CDHOME; e) and personal care (CDCARE; f) are at the bottom. It is visible that generally some AD subjects worsen their score.

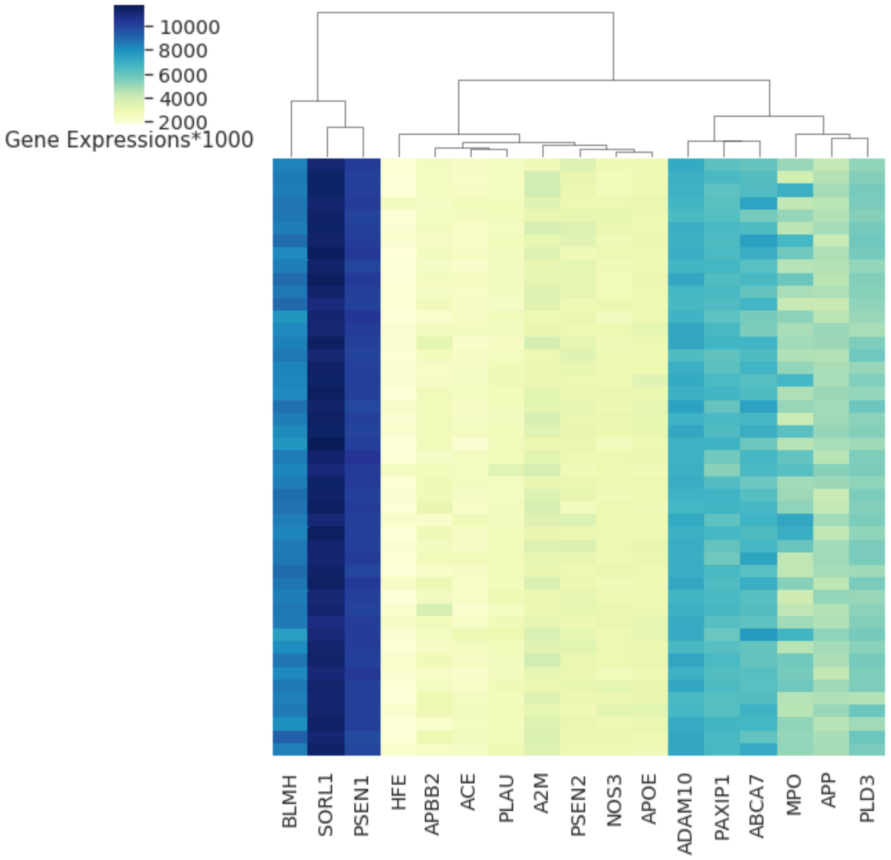

Figure S5: A heatmap of the estimated 17 gene expression profiles (values multiplied by 1000, each line represents a participant) out of the 65 probe sets as explained in the Materials and Methods section. The dark blue represents a high expression values, while the yellow represents low expression. The SORL1 has the highest expression among the genes and HFE expression was the lowest among other genes.

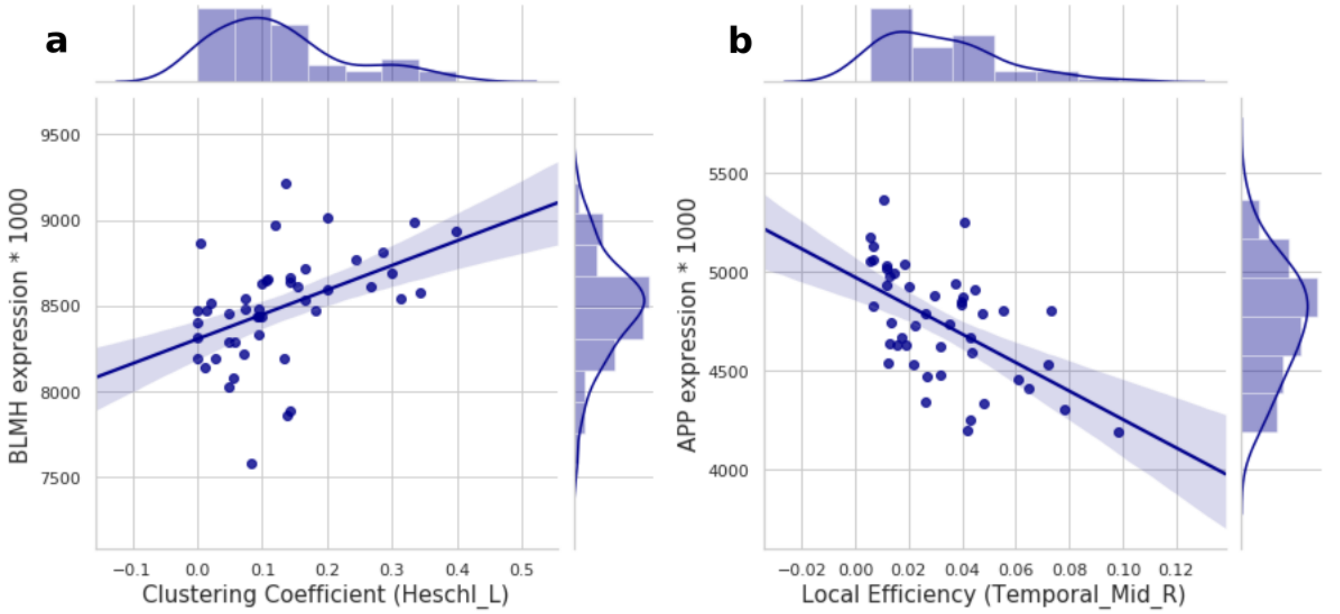

Figure S6: A scatter plot of all the significant association results. The plots shows the associations between; (a) BLMH expression and clustering coefficient in AAL region 79 (Heschl_L), (b) APP expression and local efficiency in brain region 86 (Temporal_Mid_R).

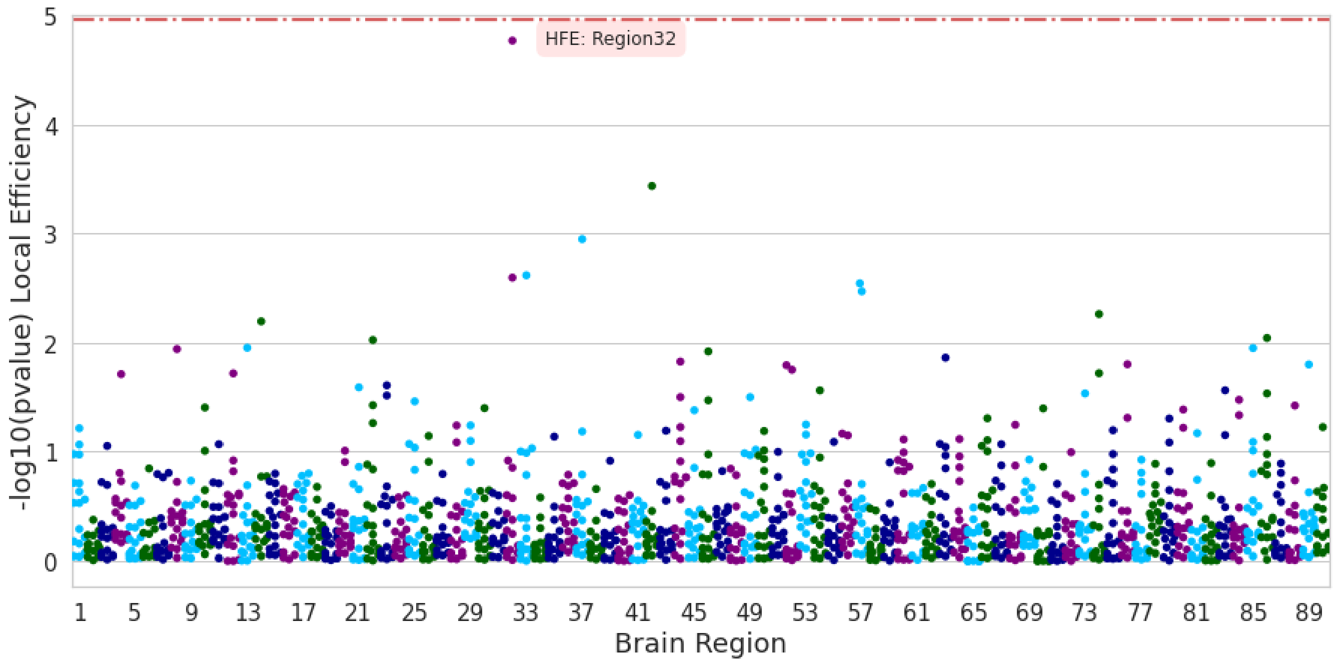

Figure S7: The figure shows the quantile regression model coefficient $-\log 10$p-values. The model regresses the change in the local coefficient (dependant variable) on a single gene at a time (independent variable), at each of the 90 brain regions as in the AAL atlas (x axis).

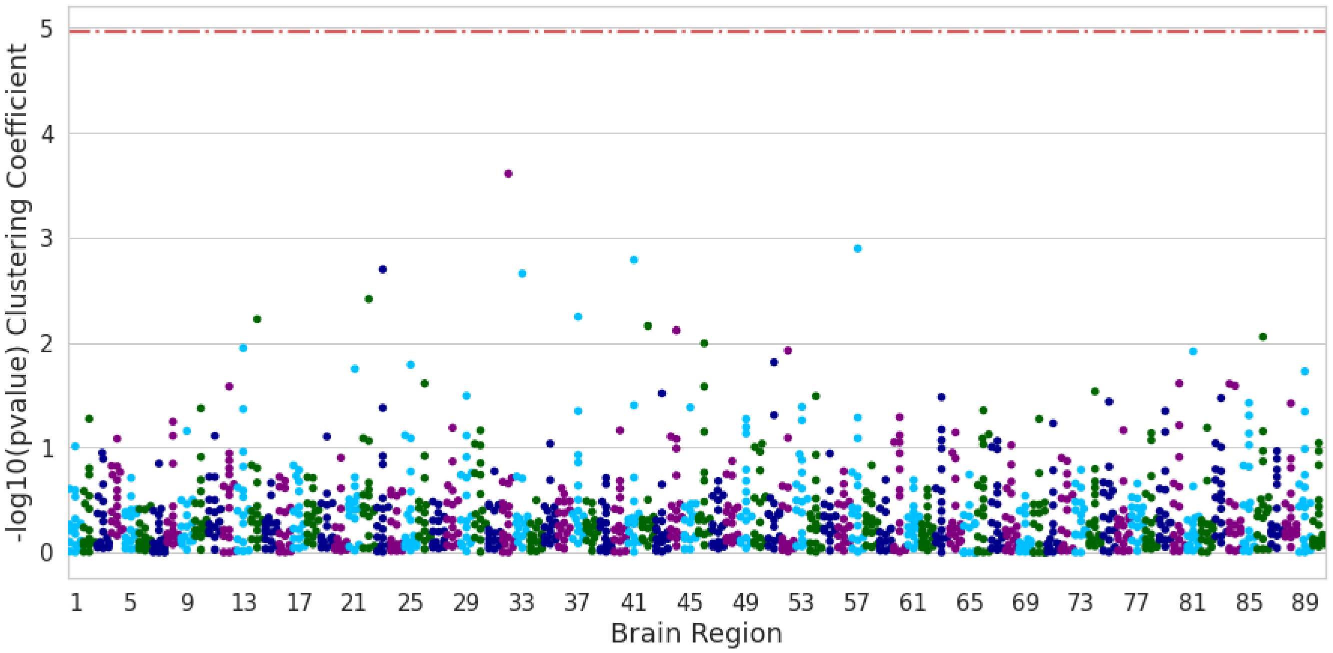

Figure S8: The figure shows the quantile regression model coefficient $-\log 10$p-values. The model regresses the change in the betweenness centrality (dependant variable) on a single gene at a time (independent variable), at each of the 90 brain regions as in the AAL atlas (x axis).

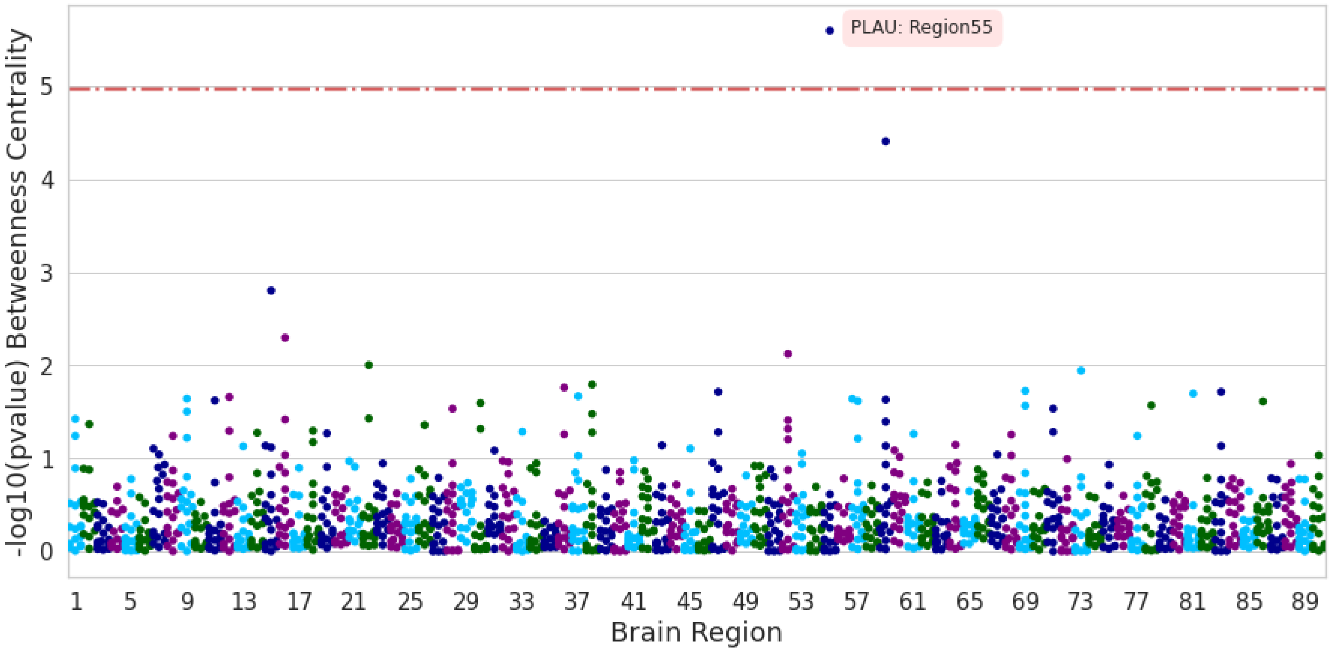

Figure S9: The figure shows the quantile regression model coefficient $-\log 10$p-values. The model regresses the change in the local coefficient (dependant variable) on a single gene at a time (independent variable), at each of the 90 brain regions as in the AAL atlas (x axis).

1.2 Tables

Table S1. Full names of brain AAL atlas regions.

**Region acronym**	Region name	Abbr.	Region id
**Precentral_L**	Precental gyrus	PreCG.L	1
**Precentral_R**	Precental gyrus	PreCG.R	2
**Frontal_Sup_L**	Superior frontal gyrus;dorsolateral	SFGdor.L	3
**Frontal_Sup_R**	Superior frontal gyrus;dorsolateral	SFGdor.R	4
**Frontal_Sup_Orb_L**	Superior frontal gyrus; orbital part	ORBsup.L	5
**Frontal_Sup_Orb_R**	Superior frontal gyrus; orbital part	ORBsup.R	6
**Frontal_Mid_L**	Middle frontal gyrus	MFG.L	7
**Frontal_Mid_R**	Middle frontal gyrus	MFG.R	8
**Frontal_Mid_Orb_L**	Middle frontal gyrus; orbital part	ORBmid.L	9
**Frontal_Mid_Orb_R**	Middle frontal gyrus; orbital part	ORBmid.R	10
**Frontal_Inf_Oper_L**	Inferior frontal gyrus;opercular part	IFGoperc.L	11
**Frontal_Inf_Oper_R**	Inferior frontal gyrus;opercular part	IFGoperc.R	12
**Frontal_Inf_Tri_L**	Inferior frontal gyrus;triangular part	IFGtriang.L	13
**Frontal_Inf_Tri_R**	Inferior frontal gyrus;triangular part	IFGtriang.R	14
**Frontal_Inf_Orb_L**	Inferior frontal gyrus; orbitalpart	ORBinf.L	15
**Frontal_Inf_Orb_R**	Inferior frontal gyrus; orbitalpart	ORBinf.R	16
**Rolandic_Oper_L**	Rolandic operculum	ROL.L	17
**Rolandic_Oper_R**	Rolandic operculum	ROL.R	18
**Supp_Motor_Area_L**	Supplementary motor area	SMA.L	19
**Supp_Motor_Area_R**	Supplementary motor area	SMA.R	20
**Olfactory_L**	Olfactory cortex	OLF.L	21
**Olfactory_R**	Olfactory cortex	OLF.R	22
**Frontal_Sup_Medial_L**	Superior frontal gyrus; medial	SFGmed.L	23
**Frontal_Sup_Medial_R**	Superior frontal gyrus; medial	SFGmed.R	24
**Frontal_Mid_Orb_L**	Superior frontal gyrus; medial orbital	ORBsupmed.L	25
**Frontal_Mid_Orb_R**	Superior frontal gyrus; medial orbital	ORBsupmed.R	26
**Rectus_L**	Gyrus rectus	REC.L	27
**Rectus_R**	Gyrus rectus	REC.R	28
**Insula_L**	Insula	INS.L	29
**Insula_R**	Insula	INS.R	30
**Cingulum_Ant_L**	Anterior cingulate and paracingulate gyri	ACG.L	31
**Cingulum_Ant_R**	Anterior cingulate and paracingulate gyri	ACG.R	32
**Cingulum_Mid_L**	Median cingulate and paracingulate gyri	DCG.L	33
**Cingulum_Mid_R**	Median cingulate and paracingulate gyri	DCG.R	34
**Cingulum_Post_L**	Posterior cingulate gyrus	PCG.L	35
**Cingulum_Post_R**	Posterior cingulate gyrus	PCG.R	36
**Hippocampus_L**	Hippocampus	HIP.L	37
**Hippocampus_R**	Hippocampus	HIP.R	38
**ParaHippocampal_L**	Parahippocampal gyrus	PHG.L	39
**ParaHippocampal_R**	Parahippocampal gyrus	PHG.R	40
**Amygdala_L**	Amygdala	AMYG.L	41
**Amygdala_R**	Amygdala	AMYG.R	42
**Calcarine_L**	Calcarine fissure and surrounding cortex	CAL.L	43
**Calcarine_R**	Calcarine fissure and surrounding cortex	CAL.R	44
**Cuneus_L**	Cuneus	CUN.L	45
**Cuneus_R**	Cuneus	CUN.R	46
**Lingual_L**	Lingual gyrus	LING.L	47
**Lingual_R**	Lingual gyrus	LING.R	48
**Occipital_Sup_L**	Superior occipital gyrus	SOG.L	49
**Occipital_Sup_R**	Superior occipital gyrus	SOG.R	50

Table S1. Full names of brain AAL atlas regions (continued).

Region acronym	Region name	Abbr.	Region id
**Occipital_Mid_L**	Middle occipital gyrus	MOG.L	51
**Occipital_Mid_R**	Middle occipital gyrus	MOG.R	52
**Occipital_Inf_L**	Inferior occipital gyrus	IOG.L	53
**Occipital_Inf_R**	Inferior occipital gyrus	IOG.R	54
**Fusiform_L**	Fusiform gyrus	FFG.L	55
**Fusiform_R**	Fusiform gyrus	FFG.R	56
**Postcentral_L**	Postcentral gyrus	PoCG.L	57
**Postcentral_R**	Postcentral gyrus	PoCG.R	58
**Parietal_Sup_L**	Superior parietal gyrus	SPG.L	59
**Parietal_Sup_R**	Superior parietal gyrus	SPG.R	60
**Parietal_Inf_L**	Inferior parietal; but supramarginal and angular gyri	IPL.L	61
**Parietal_Inf_R**	Inferior parietal; but supramarginal and angular gyri	IPL.R	62
**SupraMarginal_L**	Supramarginal gyrus	SMG.L	63
**SupraMarginal_R**	Supramarginal gyrus	SMG.R	64
**Angular_L**	Angular gyrus	ANG.L	65
**Angular_R**	Angular gyrus	ANG.R	66
**Precuneus_L**	Precuneus	PCUN.L	67
**Precuneus_R**	Precuneus	PCUN.R	68
**Paracentral_Lobule_L**	Paracentral lobule	PCL.L	69
**Paracentral_Lobule_R**	Paracentral lobule	PCL.R	70
**Caudate_L**	Caudate nucleus	CAU.L	71
**Caudate_R**	Caudate nucleus	CAU.R	72
**Putamen_L**	Lenticular nucleus; putamen	PUT.L	73
**Putamen_R**	Lenticular nucleus; putamen	PUT.R	74
**Pallidum_L**	Lenticular nucleus; pallidum	PAL.L	75
**Pallidum_R**	Lenticular nucleus; pallidum	PAL.R	76
**Thalamus_L**	Thalamus	THA.L	77
**Thalamus_R**	Thalamus	THA.R	78
**Heschl_L**	Heschl gyrus	HES.L	79
**Heschl_R**	Heschl gyrus	HES.R	80
**Temporal_Sup_L**	Superior temporal gyrus	STG.L	81
**Temporal_Sup_R**	Superior temporal gyrus	STG.R	82
**Temporal_Pole_Sup_L**	Temporal pole: superior temporal gyrus	TPOsup.L	83
**Temporal_Pole_Sup_R**	Temporal pole: superior temporal gyrus	TPOsup.R	84
**Temporal_Mid_L**	Middle temporal gyrus	MTG.L	85
**Temporal_Mid_R**	Middle temporal gyrus	MTG.R	86
**Temporal_Pole_Mid_L**	Temporal pole: middle temporal gyrus	TPOmid.L	87
**Temporal_Pole_Mid_R**	Temporal pole: middle temporal gyrus	TPOmid.R	88
**Temporal_Inf_L**	Inferior temporal gyrus	ITG.L	89
**Temporal_Inf_R**	Inferior temporal gyrus	ITG.R	90

Table S2. Quantile regression top results of regressing CDR scores on the local connectivity metrics.

**CDR**	**Results are sorted according to p-value. Threshold**= $\frac{0.05}{6\times90} = 9.26e-05$				
	Metric	Region	Region id	β	P-value
**CDJUDGE**	betweencentrality	Frontal_Inf_Oper_L	11	-1.06e-08	1.3246e-17
**CDCOMMUN**	betweencentrality	Frontal_Inf_Tri_L	13	1.162e-07	1.0377e-16
**CDCOMMUN**	betweencentrality	Pallidum_R	76	6.79e-08	1.5932e-16
**CDCARE**	betweencentrality	Pallidum_R	76	1.21e-08	2.5409e-15
**CDCARE**	betweencentrality	Frontal_Inf_Tri_L	13	-2.35e-08	4.3817e-15
**CDCARE**	betweencentrality	Rolandic_Oper_R	18	-5e-09	5.5180e-14
**CDCARE**	betweencentrality	Frontal_Mid_Orb_L	9	8.35e-08	6.8455e-14
**CDCARE**	betweencentrality	Frontal_Inf_Tri_R	14	1.538e-07	4.6868e-13
**CDMEMORY**	betweencentrality	Pallidum_R	76	9.8e-09	1.8588e-12
**CDMEMORY**	betweencentrality	Heschl_R	80	-1.431e-07	7.9135e-12
**CDHOME**	betweencentrality	Pallidum_R	76	2.34e-08	1.0339e-10
**CDORIENT**	betweencentrality	Rolandic_Oper_R	18	3.566e-07	2.8690e-10
**CDCOMMUN**	betweencentrality	Frontal_Sup_Medial_L	23	1.2e-08	2.2249e-09
**CDMEMORY**	betweencentrality	Frontal_Mid_Orb_L	9	1.53e-08	2.7792e-09
**CDHOME**	betweencentrality	Frontal_Inf_Tri_L	13	7.55e-08	2.8144e-09
**CDCARE**	local_eff	Parietal_Sup_L	59	-2.8362e-06	4.5150e-09
**CDCARE**	betweencentrality	Caudate_R	72	-2.7e-09	5.9383e-08
**CDCARE**	betweencentrality	Frontal_Sup_Medial_L	23	1.81e-08	8.4091e-08
**CDCOMMUN**	betweencentrality	Precentral_L	1	5.6e-09	8.8635e-08
**CDCARE**	betweencentrality	Frontal_Sup_Orb_L	5	-6.8e-09	9.1463e-08
**CDCARE**	betweencentrality	Occipital_Inf_L	53	3.2e-09	1.0397e-07
**CDJUDGE**	betweencentrality	Insula_R	30	-1.9e-09	1.0927e-07
**CDORIENT**	betweencentrality	Angular_R	66	-2.58e-08	1.3192e-07
**CDHOME**	betweencentrality	Frontal_Sup_Medial_L	23	7.28e-08	1.4609e-07
**CDMEMORY**	betweencentrality	Angular_L	65	9.69e-08	1.5599e-07
**CDCOMMUN**	betweencentrality	Occipital_Inf_L	53	8e-09	1.6902e-07
**CDCOMMUN**	betweencentrality	Occipital_Sup_R	50	8.8e-09	1.8204e-07
**CDCARE**	betweencentrality	Cingulum_Post_R	36	3.55e-08	2.6603e-07
**CDCARE**	betweencentrality	Angular_L	65	5.9e-09	3.2401e-07
**CDCARE**	betweencentrality	Frontal_Inf_Oper_R	12	-4.9e-09	3.9313e-07
**CDMEMORY**	betweencentrality	Frontal_Inf_Oper_R	12	1.72e-08	4.2464e-07
**CDJUDGE**	betweencentrality	Precentral_R	2	2.4e-09	4.2576e-07
**CDJUDGE**	betweencentrality	Paracentral_Lobule_R	70	5.1e-09	4.5211e-07
**CDCARE**	local_eff	Caudate_L	71	7.203e-07	6.0570e-07
**CDMEMORY**	betweencentrality	Occipital_Mid_R	52	-1.45e-08	7.3061e-07
**CDJUDGE**	betweencentrality	SupraMarginal_R	64	-1.35e-08	7.3933e-07
**CDJUDGE**	betweencentrality	Calcarine_L	43	1.2e-09	8.0399e-07
**CDCARE**	betweencentrality	Precentral_L	1	3.9e-09	1.0015e-06
**CDCARE**	local_eff	Thalamus_R	78	-1.6719e-06	1.1912e-06
**CDCARE**	betweencentrality	ParaHippocampal_L	39	8.1e-09	1.2821e-06
**CDMEMORY**	betweencentrality	Precentral_L	1	4.9e-09	1.4549e-06
**CDJUDGE**	betweencentrality	Cingulum_Mid_L	33	5e-10	1.8206e-06
**CDORIENT**	betweencentrality	Amygdala_R	42	2.7e-08	2.0376e-06
**CDCARE**	betweencentrality	Cuneus_R	46	2.6e-09	2.1374e-06
**CDJUDGE**	local_eff	Occipital_Mid_L	51	-1.3593e-06	2.5568e-06
**CDCARE**	betweencentrality	Occipital_Sup_R	50	2.4e-09	2.7811e-06
**CDCARE**	betweencentrality	SupraMarginal_R	64	2.35e-08	2.8475e-06
**CDCARE**	cluster_coef	Parietal_Sup_L	59	-1.5812e-06	3.0162e-06
**CDCARE**	betweencentrality	Precentral_R	2	6.4e-09	3.0711e-06
**CDJUDGE**	betweencentrality	Cuneus_R	46	8e-10	3.1560e-06

Table S2. Quantile regression top results of regressing CDR scores on the local connectivity metrics (continued).

**CDR**	**Results are sorted according to p-value. Threshold**$=\frac{0.05}{6\times90}=9.26e-05$				
	Metric	Region	Region id	β	P-value
**CDMEMORY**	betweencentrality	Precentral_R	2	1.21e-08	4.0628e-06
**CDCARE**	betweencentrality	Occipital_Mid_L	51	-2e-09	4.7259e-06
**CDCARE**	betweencentrality	Temporal_Inf_R	90	-8e-10	5.1879e-06
**CDCOMMUN**	betweencentrality	Temporal_Pole_Sup_R	84	1.7e-09	5.2490e-06
**CDCARE**	local_eff	Paracentral_Lobule_R	70	4.028e-07	5.3093e-06
**CDCARE**	betweencentrality	Olfactory_R	22	2.5e-09	6.1963e-06
**CDCARE**	betweencentrality	Pallidum_L	75	2.8e-09	6.6154e-06
**CDJUDGE**	betweencentrality	Postcentral_L	57	-4e-10	6.6330e-06
**CDCARE**	betweencentrality	Frontal_Med_Orb_L	25	-5.5e-09	6.7257e-06
**CDCARE**	betweencentrality	Parietal_Inf_L	61	-7.6e-09	6.8700e-06
**CDCARE**	local_eff	Calcarine_L	43	-1.3701e-06	6.9599e-06
**CDCARE**	betweencentrality	Cingulum_Mid_L	33	9e-10	7.0795e-06
**CDHOME**	betweencentrality	Precentral_L	1	6.5e-09	7.2322e-06
**CDJUDGE**	cluster_coef	Occipital_Mid_L	51	-7.789e-07	7.5340e-06
**CDCARE**	betweencentrality	Olfactory_L	21	1.2e-09	1.0177e-05
**CDCARE**	betweencentrality	Paracentral_Lobule_L	69	2.9e-09	1.0179e-05
**CDORIENT**	betweencentrality	Frontal_Sup_Medial_L	23	4.18e-08	1.1987e-05
**CDCARE**	cluster_coef	Thalamus_R	78	-9.55e-07	1.2007e-05
**CDMEMORY**	betweencentrality	Frontal_Sup_Orb_L	5	-2.3e-09	1.3418e-05
**CDCARE**	betweencentrality	Frontal_Sup_Medial_R	24	-2.2e-09	1.3753e-05
**CDMEMORY**	betweencentrality	Thalamus_L	77	4.6e-09	1.4079e-05
**CDCARE**	betweencentrality	Putamen_R	74	-6.6e-09	1.4768e-05
**CDCOMMUN**	betweencentrality	Putamen_R	74	-1.1e-09	1.7248e-05
**CDCARE**	local_eff	Amygdala_R	42	-1.2503e-06	1.7740e-05
**CDCARE**	betweencentrality	Thalamus_L	77	-7e-10	1.7820e-05
**CDJUDGE**	betweencentrality	Frontal_Inf_Orb_R	16	6e-10	1.7857e-05
**CDJUDGE**	cluster_coef	Temporal_Mid_L	85	6.814e-07	1.7954e-05
**CDJUDGE**	betweencentrality	Pallidum_L	75	6e-10	1.9036e-05
**CDCARE**	local_eff	Lingual_L	47	-1.3648e-06	1.9534e-05
**CDCARE**	betweencentrality	Putamen_L	73	-9e-10	2.0543e-05
**CDCARE**	local_eff	Frontal_Mid_Orb_L	9	-9.801e-07	2.0693e-05
**CDJUDGE**	local_eff	Temporal_Mid_L	85	1.1769e-06	2.4200e-05
**CDJUDGE**	cluster_coef	Frontal_Sup_Orb_L	5	5.54e-07	2.5902e-05
**CDCARE**	betweencentrality	Temporal_Pole_Sup_L	83	-2.4e-09	2.7526e-05
**CDJUDGE**	local_eff	Calcarine_L	43	-5.122e-07	2.7953e-05
**CDJUDGE**	cluster_coef	Cuneus_R	46	-6.206e-07	2.8360e-05
**CDCARE**	betweencentrality	Frontal_Med_Orb_R	26	1.1e-09	2.9931e-05
**CDCARE**	betweencentrality	Rectus_L	27	1.7e-09	3.1086e-05
**CDCARE**	betweencentrality	Temporal_Pole_Sup_R	84	4e-10	3.2473e-05
**CDCARE**	local_eff	Precentral_R	2	-1.7287e-06	3.3132e-05
**CDJUDGE**	local_eff	Frontal_Sup_Orb_L	5	7.069e-07	3.3295e-05
**CDCARE**	cluster_coef	Precuneus_L	67	-1.3847e-06	3.3801e-05
**CDJUDGE**	cluster_coef	Olfactory_L	21	3.541e-07	3.5295e-05
**CDCARE**	cluster_coef	Occipital_Sup_L	49	-1.2676e-06	3.5428e-05
**CDJUDGE**	cluster_coef	Calcarine_L	43	-4.54e-07	3.7033e-05
**CDJUDGE**	local_eff	Cuneus_R	46	-1.0686e-06	3.7315e-05
**CDCARE**	local_eff	Occipital_Sup_L	49	-2.3923e-06	3.9588e-05
**CDCOMMUN**	betweencentrality	Frontal_Sup_Orb_R	6	1e-09	4.0230e-05
**CDCARE**	betweencentrality	Rolandic_Oper_L	17	6.2e-08	4.0397e-05

Table S2. Quantile regression top results of regressing CDR scores on the local connectivity metrics (continued).

**CDR**	**Results are sorted according to p-value. Threshold**$=\frac{0.05}{6\times 90}=9.26e-05$				
	Metric	Region	Region id	β	P-value
**CDJUDGE**	betweencentrality	Insula_L	29	2e-10	4.3809e-05
**CDR_diff**	betweencentrality	Frontal_Sup_Medial_L	23	0.0037021312	4.5916e-05
**CDCOMMUN**	cluster_coef	Precuneus_L	67	-1.9329e-06	4.9971e-05
**CDCARE**	local_eff	Occipital_Mid_L	51	-1.4926e-06	5.3963e-05
**CDCARE**	betweencentrality	Temporal_Inf_L	89	2e-10	5.5267e-05
**CDCARE**	betweencentrality	Amygdala_L	41	-1.1e-09	5.6693e-05
**CDCARE**	betweencentrality	Frontal_Inf_Orb_L	15	-8e-10	5.6717e-05
**CDJUDGE**	betweencentrality	Putamen_R	74	3e-10	5.8176e-05
**CDCARE**	local_eff	Parietal_Inf_L	61	1.8772e-06	5.9063e-05
**CDORIENT**	local_eff	Caudate_L	71	2.6838e-06	5.9186e-05
**CDCARE**	local_eff	ParaHippocampal_L	39	-6.989e-07	6.0628e-05
**CDCARE**	cluster_coef	Paracentral_Lobule_L	69	-3.682e-07	6.0769e-05
**CDHOME**	betweencentrality	Cingulum_Mid_L	33	1.12e-08	6.2336e-05
**CDCARE**	local_eff	Temporal_Pole_Sup_R	84	-2.5008e-06	6.4671e-05
**CDCARE**	betweencentrality	Calcarine_R	44	-2.3e-09	7.4457e-05
**CDCARE**	betweencentrality	ParaHippocampal_R	40	-1.2e-09	7.5724e-05
**CDMEMORY**	local_eff	Temporal_Mid_L	85	4.7978e-06	7.5984e-05
**CDORIENT**	betweencentrality	Thalamus_L	77	3.7e-09	8.3093e-05
**CDCARE**	cluster_coef	Parietal_Inf_L	61	9.696e-07	8.3094e-05
**CDCARE**	betweencentrality	Precuneus_L	67	1.3e-09	8.9269e-05
**CDJUDGE**	local_eff	Precentral_R	2	-1.0795e-06	8.9400e-05
**CDCOMMUN**	betweencentrality	Cingulum_Mid_L	33	1.8e-09	8.9822e-05

Table S3. Ridge regression results of the change CDR scores on the global connectivity changes and Alzheimer's Disease gene expressions.

CDR	Metric	Alpha	score	Metric	*APBB2*	*MPO*	*APP*	*ACE*	*PLAU*	*PAXIP1*	*HFE*	*SORL1*	*A2M*	*NOS3*	*BLMH*	*ADAM10*	*PLD3*	*ApoE*	*PSEN1*	*PSEN2*	*ABCA7*
**CDMEMORY**	transitivity	0.1	-0.0747	-0.6704	0.122	0.0823	0.0248	-0.0392	-0.0556	0.0497	-0.0926	0.0138	-0.3465	0.3276	-0.1321	-0.1347	-0.0774	-0.2426	0.161	-0.1369	0.1903
**CDMEMORY**	global_eff	0.1	-0.0753	-0.0304	0.1114	0.0802	0.0332	-0.0311	-0.0575	0.06	-0.0765	0.0086	-0.3486	0.3226	-0.1364	-0.1318	-0.0628	-0.267	0.1585	-0.1392	0.1919
**CDMEMORY**	louvain	0.1	-0.0754	-0.259	0.1052	0.0823	0.0353	-0.0259	-0.0676	0.0647	-0.0634	0.0098	-0.3483	0.3178	-0.1388	-0.1296	-0.0616	-0.2694	0.1563	-0.1373	0.1884
**CDMEMORY**	char_path_len	0.1	-0.0752	-0.1203	0.1004	0.0804	0.0351	-0.0335	-0.0617	0.0619	-0.0769	0.0074	-0.3507	0.3187	-0.1365	-0.1287	-0.0475	-0.2827	0.1621	-0.1398	0.1869
**CDORIENT**	transitivity	0.1	-0.0677	-0.3061	0.1731	0.0679	0.2135	0.0287	0.0046	-0.0399	-0.472	0.0156	-0.192	0.0553	-0.0138	-0.0342	-0.0135	-0.1967	0.1309	-0.232	-0.0035
**CDORIENT**	global_eff	0.1	-0.0677	-0.0517	0.169	0.067	0.2172	0.0324	0.0042	-0.0354	-0.4648	0.0133	-0.1928	0.0532	-0.0157	-0.0332	-0.0078	-0.2068	0.1297	-0.2331	-0.0025
**CDORIENT**	louvain	0.1	-0.0673	-0.403	0.1592	0.0702	0.2203	0.0406	-0.0117	-0.0281	-0.4445	0.0151	-0.1923	0.0457	-0.0195	-0.0298	-0.0058	-0.2107	0.1261	-0.23	-0.0079
**CDORIENT**	char_path_len	0.1	-0.0677	0.009	0.1688	0.067	0.2173	0.0325	0.0039	-0.0352	-0.4645	0.0132	-0.1929	0.0532	-0.0158	-0.033	-0.0076	-0.2071	0.1295	-0.2331	-0.0025
**CDJUDGE**	transitivity	149	-0.0056	-0.0	-0.0002	0.0023	-0.0007	-0.0012	0.0	0.0019	0.0003	0.0002	0.0004	0.0002	-0.0005	-0.0002	-0.0007	-0.0003	-0.0005	-0.0001	-0.0027
**CDJUDGE**	global_eff	149	-0.0056	0.0	-0.0002	0.0023	-0.0007	-0.0012	0.0	0.0019	0.0003	0.0002	0.0004	0.0002	-0.0005	-0.0002	-0.0007	-0.0003	-0.0005	-0.0001	-0.0027
**CDJUDGE**	louvain	149	-0.0056	0.0001	-0.0002	0.0023	-0.0007	-0.0012	0.0	0.0019	0.0003	0.0002	0.0004	0.0002	-0.0005	-0.0002	-0.0007	-0.0003	-0.0005	-0.0001	-0.0027
**CDJUDGE**	char_path_len	149	-0.0056	-0.0001	-0.0002	0.0023	-0.0007	-0.0012	0.0	0.0019	0.0003	0.0002	0.0004	0.0002	-0.0005	-0.0002	-0.0007	-0.0003	-0.0005	-0.0001	-0.0027
**CDCOMMUN**	transitivity	0.3	-0.0325	-0.0526	0.1227	-0.0205	-0.178	0.1151	0.1812	-0.0161	-0.0366	-0.0375	-0.1628	0.1476	-0.0795	0.1688	-0.0516	0.0563	0.0078	-0.1135	0.0637
**CDCOMMUN**	global_eff	0.3	-0.0325	0.0071	0.1218	-0.0207	-0.1775	0.1154	0.1811	-0.0155	-0.0356	-0.0378	-0.1629	0.1473	-0.0798	0.1692	-0.0503	0.0547	0.0076	-0.1137	0.0637
**CDCOMMUN**	louvain	0.3	-0.0325	0.0174	0.1223	-0.0208	-0.1776	0.1152	0.1817	-0.0158	-0.0363	-0.0379	-0.1629	0.1476	-0.0797	0.169	-0.0505	0.0548	0.0077	-0.1138	0.064
**CDCOMMUN**	char_path_len	0.5	-0.0324	-0.0703	0.1038	-0.0164	-0.1436	0.0941	0.1423	-0.0117	-0.0184	-0.0381	-0.1579	0.1242	-0.0666	0.1484	-0.0458	0.0447	0.0139	-0.1111	0.0613
**CDHOME**	transitivity	0.4	-0.0893	0.0428	0.1908	-0.0343	-0.09	0.0756	0.0009	-0.0142	0.0578	-0.1228	-0.1029	-0.0044	-0.1787	0.3326	-0.095	0.1188	0.113	-0.3091	0.1076
**CDHOME**	global_eff	0.3	-0.0891	0.0872	0.1961	-0.0363	-0.0987	0.0821	-0.0017	-0.0156	0.0521	-0.132	-0.1063	-0.0057	-0.1846	0.3515	-0.0929	0.1258	0.1227	-0.3232	0.1079
**CDHOME**	louvain	0.3	-0.0892	-0.0191	0.1972	-0.0361	-0.0989	0.0826	-0.0017	-0.0157	0.0525	-0.1318	-0.1059	-0.0054	-0.1847	0.3511	-0.0949	0.1275	0.1225	-0.3231	0.1082
**CDHOME**	char_path_len	0.6	-0.0891	-0.1843	0.1674	-0.0302	-0.0767	0.0607	0.0025	-0.0114	0.0616	-0.1083	-0.0978	-0.0083	-0.167	0.3047	-0.0782	0.0932	0.1009	-0.2832	0.0988
**CDCARE**	transitivity	149	-0.1093	-0.0	-0.0008	0.0027	-0.0014	-0.0002	0.0007	0.0001	-0.0037	0.0013	-0.0066	-0.001	-0.0037	-0.0018	-0.0031	0.0	0.0013	-0.0045	0.0078
**CDCARE**	global_eff	149	-0.1093	-0.0	-0.0008	0.0027	-0.0014	-0.0002	0.0007	0.0001	-0.0037	0.0013	-0.0066	-0.001	-0.0037	-0.0018	-0.0031	0.0	0.0013	-0.0045	0.0078
**CDCARE**	louvain	149	-0.1093	-0.0001	-0.0008	0.0027	-0.0014	-0.0002	0.0007	0.0001	-0.0037	0.0013	-0.0066	-0.001	-0.0037	-0.0018	-0.0031	0.0	0.0013	-0.0045	0.0078
**CDCARE**	char_path_len	149	-0.1093	0.0	-0.0008	0.0027	-0.0014	-0.0002	0.0007	0.0001	-0.0037	0.0013	-0.0066	-0.001	-0.0037	-0.0018	-0.0031	0.0	0.0013	-0.0045	0.0078
**CDR_diff**	transitivity	0.1	-1.2761	-1.5541	0.7401	0.1734	-0.2195	0.1158	0.197	0.0516	-0.8229	-0.0517	-1.2177	0.8435	-0.6461	0.3132	-0.3519	-0.337	0.6769	-1.3473	0.6318
**CDR_diff**	global_eff	0.1	-1.2782	0.1652	0.7112	0.1687	-0.1988	0.134	0.1901	0.0768	-0.7842	-0.0645	-1.2239	0.8302	-0.6564	0.3216	-0.3117	-0.3999	0.6719	-1.353	0.634
**CDR_diff**	louvain	0.1	-1.28	-0.4664	0.7041	0.1724	-0.1962	0.1439	0.1741	0.084	-0.762	-0.0618	-1.2221	0.823	-0.6605	0.3239	-0.3153	-0.3984	0.6672	-1.3492	0.6292
**CDR_diff**	char_path_len	0.1	-1.2776	-1.0277	0.6252	0.1706	-0.1849	0.1144	0.1581	0.0904	-0.7903	-0.0733	-1.2397	0.8002	-0.6563	0.3454	-0.1921	-0.5225	0.7013	-1.3571	0.5943
